# Steep rise in norovirus cases and emergence of a new recombinant strain GII.P16-GII.2, Germany, winter 2016

**DOI:** 10.2807/1560-7917.ES.2017.22.4.30447

**Published:** 2017-01-26

**Authors:** S Niendorf, S Jacobsen, M Faber, A M Eis-Hübinger, J Hofmann, O Zimmermann, M Höhne, C T Bock

**Affiliations:** 1 Department of Infectious Diseases, Robert Koch-Institute, Berlin, Germany; 2 Consultant Laboratory for Noroviruses, Robert Koch-Institute, Berlin, Germany; 3 Department for Infectious Disease Epidemiology, Robert Koch-Institute, Berlin, Germany; 4 Institute of Virology, University of Bonn Medical Centre, Bonn, Germany; 5 Institute of Medical Virology, Charité University Medicine, Berlin, and Labor Berlin, Charité-Vivantes GmbH, Berlin, Germany; 6 Institute of Medical Microbiology, Göttingen, Germany

**Keywords:** norovirus, recombinant, GII.P16-GII.2, emergence, Germany

## Abstract

Since early November 2016, the number of laboratory-confirmed norovirus infections reported in Germany has been increasing steeply. Here, we report the detection and genetic characterisation of an emerging norovirus recombinant, GII.P16-GII.2. This strain was frequently identified as the cause of sporadic cases as well as outbreaks in nine federal states of Germany. Our findings suggest that the emergence of GII.P16-GII.2 contributed to rising case numbers of norovirus gastroenteritis in Germany.

In 2016, the increase of notified norovirus cases in the winter season was unexpectedly strong and early (Figure 1) in Germany. In November 2016, 14,872 laboratory-confirmed cases were reported to the national public health authority compared with a median of 7,810 cases in the same month of the previous five years. This may be due to a new variant’s ability to escape herd immunity to the previously circulating strains. In this study, we conducted a phylogenetic analysis of the currently circulating norovirus strains in order to assess whether one or several new strains could be responsible for the current steep rise in norovirus cases.

**Figure 1:**
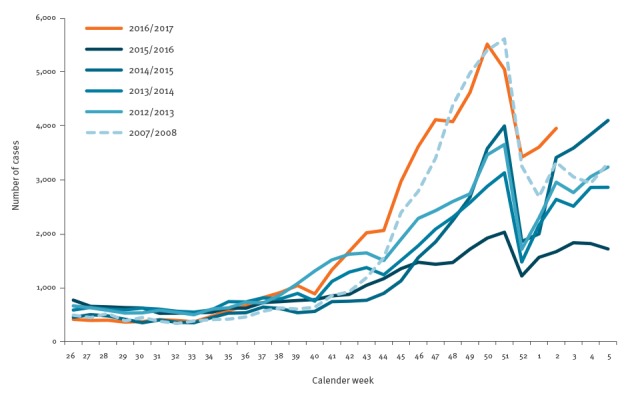
Laboratory-confirmed norovirus infections by calendar week and year of notification, compared previous seasons, Germany, week 26 2016–week 2 2017 (n = 56,384)

Data as on 24 Jan 2017.

## Sample collection and molecular characterisation

The Consultant Laboratories (CL) and National Reference centre (NRC) are officially appointed and funded by the German Federal Ministry of Health and play a central role in detection and prevention of infection disease in Germany. The coordination of the CL and NRC is hosted by the Robert Koch-Institute. The CL for norovirus at the Robert Koch-Institute is focused on the molecular surveillance of viral gastroenteritis pathogens, especially noroviruses. For genotyping analysis, stool specimens from norovirus-positive outbreaks were sent to the CL by diagnostic laboratories, physicians and local public health authorities. Between September and December 2016, 240 norovirus positive stool samples from patients with norovirus-associated AGE from 13 federal states of Germany were analysed at the CL for noroviruses. Altogether 175 samples were associated with 69 outbreaks, mainly in childcare facilities (n = 39 outbreaks) and nursing homes (n = 12 outbreaks) in 11 of 16 federal states (Baden-Wuerttemberg, Bavaria, Berlin, Hesse, Lower Saxony, Mecklenburg-Western Pomerania, North Rhine-Westphalia, Rhineland-Palatinate, Saxony, Schleswig-Holstein and Thuringia). Altogether 65 samples were from sporadic AGE and were sent by hospitals and diagnostics laboratories from six federal states (Baden-Wuerttemberg, Berlin, Brandenburg, Hamburg, Lower Saxony and North Rhine-Westphalia). 

Samples were genotyped as previously described [6] by phylogenetic analysis of ORF1 and ORF2 sequences. To determine the recombination breakpoint, 14 samples of the new norovirus recombinant were analysed in addition, using a newly established semi-nested RT-PCR spanning the 3’ end of the ORF1 and the P2 domain. In brief, RT-PCR reactions were performed using SuperScriptIII One-Step RT-PCR system Platinum TAQ DNA Polymerase (Thermo Fisher, Walthman MA, US) and primer sets NV1a (5’-ATGAATATGAATGAAGATGG-3’), NV1b (5’-ATGAACACAATAGAAGATGG-3’), NV348a (5’-GGTTRACCCARGAATCAAA-3’), NV348b (5’-GRTTMACCCAAGAITCAAA-3’) and NV348c (5’-GRTTRACCCAIACTTCAAA-3’) for the first PCR (2328 bp fragment). The second PCR reaction was carried out using the HotStarTaq Master Mix Kit (Qiagen, Hildesheim, Germany) and primers NV6 (5’-TACCACTATGATGCAGATTA-3’), NV6a (5’-TATCACTATGATGCTGACTA-3’), NV348a, NV348b, NV348c. PCR conditions were 5 min at 55 °C, 55 min at 45 °C, 2 min at 94 °C, followed by 40 cycles of 15 s at 94 °C, 30 s at 45 °C, 3 min at 68 °C and finally 5 min at 68 °C. The resulting 2,274 bp amplicons were subjected to direct sequencing. Nucleotide sequences of these samples were submitted to the GenBank database with the accession numbers KY357449 to KY357462.

## Molecular genetic results

We identified emerging recombinant norovirus strains previously not described in Germany in outbreaks or in sporadic cases of AGE. Typing results of all 240 analysed samples are shown in the Table. 

**Table:** Distribution of norovirus genotypes detected in samples sent to the Consultant Laboratory (outbreaks vs. sporadic cases of acute gastroenteritis), Germany, September–December 2016 (n = 65 samples from sporadic cases, n = 175 samples from 69 outbreaks)

Norovirus genotype	Sporadic cases		Outbreaks	
	**n**	**%**	**n**	**%**
GI.P1-GI.1	0	0	1	1.4
GI.P3-GI.3	3	4.6	2	2.9
GI.P4-GI.4	1	1.5	3	4.3
GI.P5-GI.5	0	0	1	1.4
GI.Pb-GI.6	0	0	2	2.9
GII.P2-GII.2	1	1.5	0	0
GII.P4 2009-GII.4 2012	5	7.7	7	10.1
GII.P7-GII.6	1	1.5	4	5.8
GII.P7-GII.7	0	0	3	4.3
GII.P8-GII.8	0	0	1	1.4
**GII.P16-GII.2**	**31**	**47.7**	**29**	**42.0**
GII.P16-GII.4 2012	7	10.8	7	10.1
GII.P17-GII.17	0	0	6	8.7
GII.P21-GII.3	2	3.1	1	1.4
GII.P21-GII.13	1	1.5	0	0
GII.Pe-GII.4 2012	12	18.5	2	2.9
GII.Pg-GII.1	1	1.5	0	0
**Total**	**65**	**100**	**69**	**100**

**Figure 2:**
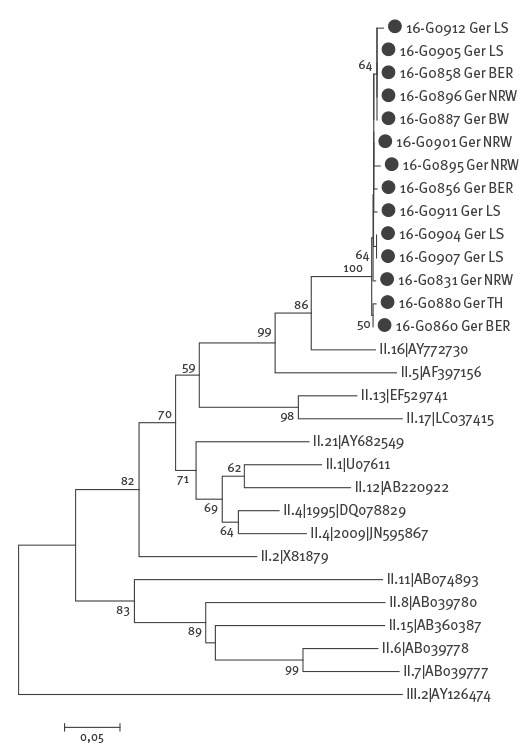
Phylogenetic analysis based on the nucleotide sequence of a 357 bp region (ORF1) of genogroup II norovirus, Germany, 2016/17 (n = 14 representative samples)

BER: Berlin; BW: Baden-Wuerttemberg; Ger: Germany; LS: Lower Saxony; NRW: North Rhine-Westphalia; TH: Thuringia.

**Figure 3:**
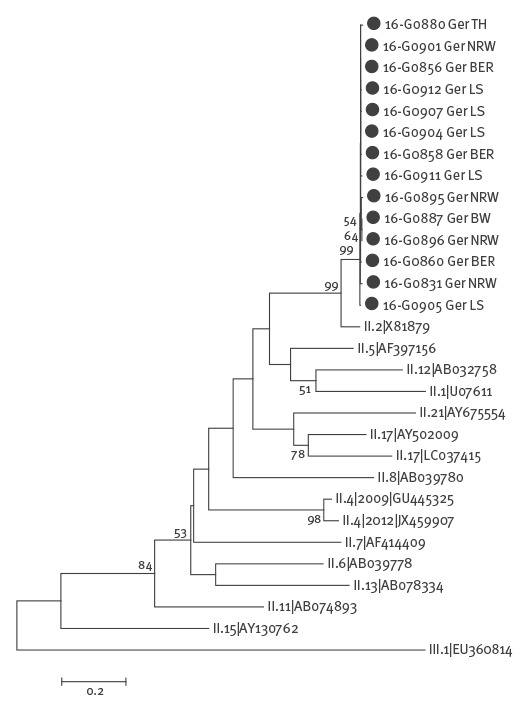
Phylogenetic analysis based on the nucleotide sequence of a 628 bp region of the P2 domain (ORF2) of genogroup II noroviruses, Germany, 2016/17 (n = 14 representative samples)

BER: Berlin; BW: Baden-Wuerttemberg; Ger: Germany; LS: Lower Saxony; NRW: North Rhine-Westphalia; TH: Thuringia.

The phylogenetic analysis revealed a recombination of GII.P16 (ORF1) and GII.2 (ORF2) strains (Figures 2 and 3). Using SimPlot analysis, the recombination point could be mapped to the ORF1/ORF2 junction region at nucleotide positions 732–734 (data not shown). The recombinant strain GII.P16-GII.2 was detected in 29 of 69 investigated outbreaks, in nine of the 11 federal states of Germany that had outbreaks (Baden-Wuerttemberg, Bavaria, Berlin, Hesse, Lower Saxony, Mecklenburg-Western Pomerania, North Rhine-Westphalia, Rhineland-Palatinate and Thuringia) and was considered as the aetiological agents in 31 of 65 cases of sporadic AGE. The new recombinant was detected in specimens obtained from the sporadic cases in four hospitals in Berlin, North Rhine-Westphalia, Baden-Wuerttemberg and Lower Saxony. Besides the new recombinant strain, the well-known norovirus genotypes GI.P3-GI.3 and GII.P17-GII.17 and the recombinant strains GII.Pe-GII.4 2012 and GII.P4 2009-GII.4 2012 were found co-circulating, were but less frequently detected in the current season.

Analysed sequence: nucleotide positions 4,332–4,689 according to accession no: AY772730. Strains analysed in this study are denoted with a black bullet (•). Scale bar indicates nucleotide substitution per site. Sequence alignments were performed with the ClustalW algorithm. Neighbour-joining phylogenetic tree was produced using the MEGA 7 software with bootstrap test (1,000 replicates). Bootstrap values (1,000 replicates) above 50 are shown. The evolutionary distances were computed using the Kimura-2 parameter method.

Analysed sequence: nucleotide positions 5,871–6,509 according to accession no: AY772730. Strains analysed in this study are denoted with a black bullet (•). Scale bar indicates nucleotide substitution per site. Sequence alignments were performed with the ClustalW algorithm. Neighbour-joining phylogenetic tree was produced using the MEGA 7 software with bootstrap test (1,000 replicates). Bootstrap values (1,000 replicates) above 50 are shown. The evolutionary distances were computed using the Kimura-2 parameter method.

## Discussion

We found a new norovirus strain GII.P16-GII.2 in samples from sporadic AGE and from norovirus outbreaks derived from nine federal states of Germany. It was recently shown that the emergence of new GII.4 norovirus variants can result in an increasing number of reported norovirus infection [5]. This has already been observed in Germany in the season 2007/08 which was also characterised by an early rise and high total number of notified norovirus infections, with most of the analysed outbreaks caused by the new epidemic variant GII.4 2006b [7]. Another example is the emergence of a novel variant GII.P17-GII.17 in the season 2014/15, which was first genotyped in China and Japan and replaced the previously dominant genotype GII.Pe-GII.4 2012 with an increased outbreak activity [8,9]. The new 2016 GII.P16-GII.2 recombinant has sporadically been reported to the international molecular surveillance database NoroNet from Australia, Finland, France and Russia, and previously from Japan and China [10,11], suggesting a worldwide distribution.

So far, it is unclear whether the new recombinant is associated with more severe symptoms. Further molecular and epidemiological investigations are needed to assess whether the emerging new recombinant norovirus strain GII.P16-GII.2 can replace the predominant GII.Pe-GII.4 2012 strain and how this will affect outbreak sizes, course of disease and herd immunity of the population, not only in Germany but also in other countries in Europe.

## References

[1] Ahmed SM Hall AJ Robinson AE Verhoef L Premkumar P Parashar UD Global prevalence of norovirus in cases of gastroenteritis: a systematic review and meta-analysis. Lancet Infect Dis. 2014;14(8):725-30. 10.1016/S1473-3099(14)70767-4 24981041 PMC8006533

[2] Kroneman A Vega E Vennema H Vinjé J White PA Hansman G Proposal for a unified norovirus nomenclature and genotyping. Arch Virol. 2013;158(10):2059-68. 10.1007/s00705-013-1708-5 23615870 PMC5570552

[3] de Graaf M van Beek J Vennema H Podkolzin AT Hewitt J Bucardo F Emergence of a novel GII.17 norovirus – End of the GII.4 era? Euro Surveill. 2015;20(26):21178. 10.2807/1560-7917.ES2015.20.26.21178 26159308 PMC5921880

[4] Bull RA Eden JS Rawlinson WD White PA Rapid evolution of pandemic noroviruses of the GII.4 lineage. PLoS Pathog. 2010;6(3):e1000831. 10.1371/journal.ppat.1000831 20360972 PMC2847951

[5] Bruggink L Catton M Marshall J A norovirus intervariant GII.4 recombinant in Victoria, Australia, June 2016: the next epidemic variant? Euro Surveill. 2016;21(39):30353. 10.2807/1560-7917.ES.2016.21.39.30353 27719750 PMC5069427

[6] Höhne M Niendorf S Mas Marques A Bock CT Use of sequence analysis of the P2 domain for characterization of norovirus strains causing a large multistate outbreak of norovirus gastroenteritis in Germany 2012. Int J Med Microbiol. 2015;305(7):612-8. 10.1016/j.ijmm.2015.08.010 26341330

[7] Bernard H Höhne M Niendorf S Altmann D Stark K Epidemiology of norovirus gastroenteritis in Germany 2001-2009: eight seasons of routine surveillance. Epidemiol Infect. 2013;142(1):63-74. 10.1017/S0950268813000435 23517686 PMC9152553

[8] Han J Ji L Shen Y Wu X Xu D Chen L Emergence and predominance of norovirus GII.17 in Huzhou, China, 2014-2015. Virol J. 2015;12(1):139. 10.1186/s12985-015-0370-9 26362650 PMC4566299

[9] Matsushima Y Ishikawa M Shimizu T Komane A Kasuo S Shinohara M Genetic analyses of GII.17 norovirus strains in diarrheal disease outbreaks from December 2014 to March 2015 in Japan reveal a novel polymerase sequence and amino acid substitutions in the capsid region. Euro Surveill. 2015;20(26):21173. 10.2807/1560-7917.ES2015.20.26.21173 26159307

[10] Iritani N Kaida A Abe N Kubo H Sekiguchi J Yamamoto SP Detection and genetic characterization of human enteric viruses in oyster-associated gastroenteritis outbreaks between 2001 and 2012 in Osaka City, Japan. J Med Virol. 2014;86(12):2019-25. 10.1002/jmv.23883 24415518

[11] Wang YH Zhou DJ Zhou X Yang T Ghosh S Pang BB Molecular epidemiology of noroviruses in children and adults with acute gastroenteritis in Wuhan, China, 2007-2010. Arch Virol. 2012;157(12):2417-24. 10.1007/s00705-012-1437-1 22886184

